# CircPOSTN/miR-361-5p/TPX2 axis regulates cell growth, apoptosis and aerobic glycolysis in glioma cells

**DOI:** 10.1186/s12935-020-01454-x

**Published:** 2020-08-06

**Authors:** Niya Long, Liangzhao Chu, Jun Jia, Shuo Peng, Yuan Gao, Hua Yang, Yaoming Yang, Yan Zhao, Jian Liu

**Affiliations:** 1 Department of Pathology, Guizhou Medical University, No. 9 Beijing Road, Guiyang, 550004 Guizhou China; 2 Key Laboratory of Endemic and Ethnic Diseases, (Guizhou Medical University) Ministry of Education, No. 9 Beijing Road, Guiyang, 550004 Guizhou China; 3 Key Laboratory of Molecular Biology, Guizhou Medical University, Guiyang, Guizhou 550004 China; 4 Department of Neurosurgery, Affiliated Hospital of Guizhou Medical University, Guiyang, Guizhou China; 5 Department of Biology, Guizhou Medical University, No. 9 Beijing Road, Guiyang, 550004 Guizhou China

**Keywords:** Circular RNA, circPOSTN, miR-361-5p, TPX2, Glioma, Aerobic glycolysis

## Abstract

**Background:**

Glioma is the most primary central nervous system tumor in adults. The 5 year survival rate for glioma patients remains poor, although treatment strategies had improved in the past few decades. The cumulative studies have shown that circular RNA (circRNA) is associated with glioma process, so the purpose of this study is to clarify the function of circPOSTN in glioma.

**Methods:**

The expression levels of circPOSTN, miR-361-5p, and targeting protein for Xenopus kinesin-like protein 2 (TPX2) were assessed with real-time quantitative polymerase chain reaction (RT-qPCR). The 3-(4, 5-dimethylthiazol-2-yl)-2, 5-diphenyl-2H-tetrazol-3-ium bromide (MTT) and flow cytometry assays were executed to examine proliferation and apoptosis of glioma cells, respectively. Western blot was applied to assess protein expression. The glucose metabolism of glioma cells was analyzed by testing the glucose consumption, lactate production, ATP level, reactive oxygen species (ROS) accumulation and performing Seahorse XF assay. The interaction relationship between miR-361-5p and circPOSTN or TPX2 was analyzed by bioinformatics database and dual-luciferase reporter assay. The influences of circPOSTN silencing in vivo were observed by a xenograft experiment.

**Results:**

CircPOSTN was overexpressed in glioma tissues and cells. Absence of circPOSTN in glioma cells promoted apoptosis while impeded proliferation and aerobic glycolysis, which were mitigated by silencing miR-361-5p. What’s more, loss-of-functional experiment suggested that knockdown of TPX2 repressed proliferation and aerobic glycolysis, while induced apoptosis in glioma cells. In addition, circPOSTN targetedly regulated TPX2 expression in glioma cells via sponging miR-361-5p. In vivo study revealed that deficiency of circPOSTN restrained tumor growth.

**Conclusion:**

Mechanistically, circPOSTN regulated cell growth, apoptosis, and aerobic glycolysis in glioma through miR-361-5p/TPX2 axis.

## Highlights

1. CircPOSTN was overexpressed in glioma tissues and cells.
2. Knockdown of circPOSTN or TPX2 repressed aerobic glycolysis in glioma cells.
3. CircPOSTN mediated proliferation, apoptosis, and aerobic glycolysis in glioma cells by regulating miR-361-5p/TPX2 axis.

## Background

Glioma, originated from the normal glial cells, is the most common type of lethal intracranial tumors with poor outcome [1]. Glioblastoma multiforme (GBM) is the most aggressive type of glioma. In addition, approximately half of patients with GBM died within 15 months [2]. Recurrence and highly invasive nature are considered as major obstacles for conquering this deadly disease. Therefore, a better understanding of the pathology of gliomagenesis and new therapeutic approaches for glioma are urgently needed.

Recently, accumulating evidence indicated that circRNA was differentially expressed and regulated tumorigenesis in human cancers [3]. CircPOSTN, located at chr13:38136718–38161065, has 2656 nucleotides in length. A study on glioma reported that circPOSTN is a novel oncogenic gene in glioma and closely relates to the poor prognosis of glioma patients [4]. However, the relationships between circPOSTN and aerobic glycolysis of glioma cells remain unknown. By the way, this metabolic reprogramming, known as the Warburg effect or aerobic glycolysis, plays significant effects on cellular metabolism and growth control in multiple cancer cells [5].

Emerging evidence indicated that miR-361-5p could act an important regulatory role in non-small cell lung cancer [6], hepatocellular carcinoma [7], and breast cancer [8]. For instance, a previous study demonstrated that miR-361-5p was an oncogenic gene in cervical cancer [9]; however, in glioma, it turned out to be a tumor suppressor miRNA that hampered cell growth and metastasis [10]. Nevertheless, the regulatory function of miR-361-5p on aerobic glycolysis of glioma cells are unknown.

As previous research had reported that targeting protein for Xenopus kinesin-like protein 2 (TPX2) could promote glioma progression by activating the protein kinase B (AKT) signaling pathway [11]. In addition, TPX2, a microtubule-associated protein, could regulate mitotic spindle through regulation of microtubule flux, suggesting the important function of TPX2 in cell cycle process [12]. TPX2 was considered as a supposed oncogene and was abnormally expressed in gastric cancer, pancreatic cancer, and colon cancer [13–15]. Furthermore, TPX2 was an independent marker for the metastasis and prognosis of esophageal cancer patients [16]. Analogously, upregulation of TPX2 positively correlated with poor prognosis in human lung squamous cell carcinoma and prostate cancer [17, 18].

Based on above studies, we measured the abundance of circPOSTN, miR-361-5p and TPX2 in glioma tissues and cells. Moreover, bioinformatics analysis and functional experiments were used to probe target relationship among them. Accordingly, the study was committed to probing the mechanism of circPOSTN/miR-361-5p/TPX2 axis on glioma.

## Materials and methods

### Human tissue sample collection

This research got permission from the Ethics Committee of Guizhou Medical University, and written the informed consents were provided by each patient or volunteer. A total of 25 patients with glioma were recruited in this study took surgically resected at Guizhou Medical University. Another 25 volunteers were recruited in this study as control. The excised glioma tissues and normal brain tissues were collected and immediately frozen in liquid nitrogen, then transferred to − 80 °C refrigerator.

### Cell culture

Normal human astrocytes (NHA) were derived from ScienCell (San Diego, CA, USA). Glioma cell line (U251) was acquired from Type Culture Collection of the Chinese Academy of Sciences (Shanghai, China). Glioma cell line (LN229) was bought from the American Type Culture Collection (Rockville, MD, USA). NHA, LN229 and U251 cells were propagated in the Dulbecco’s modified Eagle medium (GIBCO BRL, Grand Island, NY, USA) plus 10% (v/v) fetal bovine serum (FBS; GIBCO BRL), and antibiotics (penicillin/streptomycin; Invitrogen, Carlsbad, CA, USA) in an incubator at constant temperature of 37 °C with 5% CO_2_.

### Real-time quantitative polymerase chain reaction (RT-qPCR)

Total RNA extraction was performed with trizol reagent (Takara, Dalian, China). In addition, the total RNA was quantified under Nanodrop 2000c (Thermo Fisher Scientific, Carlsbad, CA, USA). For detection gene expression, complementary DNA (cDNA) was synthesized with Prime Script RT Reagent kit (Takara) and All-in-One miRNA cDNA Synthesis Kit (Invitrogen) based on the operation manual. Afterwards, RT-qPCR assay was conducted with SYBR Premix Ex Taq II (Takara) under ABI 7500 HT system (Applied Biosystems, Foster City, CA, USA). The expression level of gene was standardized to glyceraldehyde-3-phosphate dehydrogenase (GAPDH) or endogenous small nuclear RNA U6 based on the 2^−ΔΔCt^ method.

### Transfection assay

Specific small interfering RNA (siRNA) against circPOSTN (si-circPOSTN) or against TPX2 (si-TPX2) and siRNA scrambled control (si-NC), plasmid-mediated pcDNA and pcDNA-circPOSTN, miR-361-5p mimic and miR-NC, anti-miR-361-5p and anti-miR-NC were purchased from Genepharma (Shanghai, China). The lentiviral vectors of specific short hairpin RNA (shRNA) target circPOSTN (sh-circPOSTN) and shRNA scrambled control (sh-NC) were constructed by Sangon (Shanghai, China). For transfection assay, LN229 and U251 cells were seeded into 6-well plates (3 × 10^4^ cells/well) overnight, and cell density reached 70–80% prior to transfection. The transfection analysis was conducted with Lipofectamine 2000 (Invitrogen) according to the recommendations. In addition, the concentrations of oligonucleotides for transfection were 50 nM. The concentrations of plasmids for transfection were 100 nM.

### 3-(4, 5-dimethylthiazol-2-yl)-2, 5-diphenyl-2H-tetrazol-3-ium bromide (MTT) assay

The cells activity of LN229 and U251 cells at indicated time points was recorded by MTT assay. The transfected 3 × 10^3^ LN229 or U251 cells were added into each well of the 96-well plate. After incubation for indicated time, 20 μL of the MTT solution (Beyotime, Jiangsu, China) was dropped into each well of plate. After 4 h of incubation, the supernatant liquid was replaced with 150 μL of dimethyl sulfoxide in per well. After shocking for 15 min, the absorbance of each well at a reference of 490 nm was examined with a microplate spectrophotometer (BioTek Instruments, Winooski, VT, USA).

### Flow cytometry

Apoptosis rate was calculated with Annexin V-fluorescein isothiocyanate (FITC)/Propidium Iodide (PI) kit (TransGen Biotech, Beijing, China). LN229 and U251 cells were collected by centrifuging at 1000 rpm for 5 min, and then cells were rinsed with phosphate buffer saline to remove residual medium. After the supernatant was removed, the 400 μL of staining buffer contained FITC and PI was used to stain the cells at room temperature in dark condition. The flow cytometry (Applied Biosystems) was recruited to monitor cell apoptosis.

### Western blot assay

The Radio-Immunoprecipitation assay (RIPA) buffer (Cell Signal Technology, Danvers, MA, USA) was used to prepare cell or tissue proteins as per user’s guidebook. The equal amounts of proteins (30 μg) were split by 12% of sodium dodecyl sulfate polyacrylamide gel electrophoresis and then electroblotted onto nitrocellulose membranes (Millipore, Billerica, MA, USA). Next, the blocking of membranes was performed by adding 5% of dry skim milk in tris-buffered saline with Tween-20. The membranes were reacted with monoclonal antibodies overnight at 4 °C. After being washed by tris‐buffered saline with Tween‐20, membranes were incubated with horseradish peroxidase-conjugated secondary antibodies (1:1000 dilution, Boster, Wuhan, China) for 2 h. Protein binds were visualized and analyzed using enhanced chemiluminescence reagent (Pierce Biotechnology, Rockford, IL, USA) and Image Lab software 5.2 (Bio-Rad, Hercules, CA, USA), respectively. The primary antibodies containing anti-B cell lymphoma-2 (Bcl-2; 1:800 dilution, Boster), anti-Bcl-2-associated X protein (Bax;1:800 dilution, Boster), anti-hexokinase 2 (HK2; 1:800 dilution, Boster), anti-lactate dehydrogenase A (LDHA;1:800 dilution, Boster), anti-TPX2 (TPX2; 1:800 dilution, Boster), and anti-GAPDH (1:800 dilution, Boster).

### Detection of caspase-3 activity

A caspase-3 assay kit (Abcam, Cambridge, MA, USA) was utilized for analysis of the activity of caspase-3. At 48 h after transfection, LN229 and U251 cells were lysed using RIPA buffer (Cell Signal Technology) and high-centrifuged at 10,000×*g* for 3 min. Subsequently, Reaction Buffer (containing acetyl-Asp-Glu-Val-Asp *p*-nitroanilide substrate) was added to each sample and incubated at 37 °C for 30 min. The absorbance of samples was read at 405 nm under a microplate reader (Applied Biosystems). The activity of caspase-3 was analyzed based on a standard calibration curve and protein concentration.

### Glucose metabolism assay

LN229 and U251 cells transfected with indicated plasmids or oligonucleotides were injected into 12-well plates (2 × 10^4^ cells/well), and then cells were incubated in 1.5 mL of Dulbecco’s modified Eagle medium. After 48 h, partial culture medium for each well was collected for assay of glucose consumption and lactate production by glucose assay Kit (Biovision, Milpitas, CA, USA) and lactic acid assay kit (Sigma-Aldrich Chemical Company, Louis, Missouri, USA), respectively. In addition, LN229 and U251 cells were lysed for measurement LDHA enzyme activity, intracellular ATP, and ROS level by Lactate dehydrogenase activity detection kit (Sigma-Aldrich Chemical Company), ATP assay kit (Solarbio, Beijing, China), and reactive oxygen species assay kit (Solarbio), individually. All assay kit was performed in line with the producer’s direction.

### Dual-luciferase reporter assay

The bioinformatics analysis tool StarBase3.0 (http://starbase.sysu.edu.cn/) was used to predict binding sites between miR-361-5p and circPOSTN or 3′untranslated region (UTR) of TPX2. Wild-type circPOSTN-WT or TPX2 3′UTR-WT, containing complementary sequences with miR-361-5p, and circPOSTN-MUT or TPX2 3′UTR- MUT were cloned into the luciferase vector (Promega, Madison, WI, USA). The above vectors were co-transfected into LN229 and U251 cells with miR-361-5p or miR-NC according to the experimental design. The Luciferase Assay System (Promega) was used to assess luciferase activity of harvested cells after transfection 48 h.

### Glycolysis stress test

Extracellular acidification rate (ECAR) and oxygen consumption rate (OCR) analyses were carried out on Seahorse XF96 Glycolysis Analyzer (Agilent Technologies, Suzhou, China) as per user’s guidebook. ECAR and OCR were key parameters for glycolysis. For ECAR assay, glucose, oligomycin, and 2-deoxyglucose were sequentially added in medium at 25 min, 55 min, and 85 min, respectively. For OCR assay, oligomycin, Carbonyl cyanide 4-(trifluoromethoxy) phenylhydrazone (FCCP), rotenone, and antimycin were added in medium at 25 min, 55 min, and 85 min, correspondingly.

### In vivo experiment

LN229 cells were stably transfected lentiviral vectors contained sh-circPOSTN or sh-NC. The transfected LN229 cells (5 × 10^6^ cells in 100 μL of PBS) were subcutaneously injected left axillary region of male BALB/c nude mice (4 weeks of age, Shanghai Experimental Animal Center, Shanghai, China). Animals were assigned to two groups; sh-NC group and sh-circPOSTN group. The tumor volumes of mice were examined at 7 days, 11 days, 15 days, 19 days, 23 days, and 27 days post-injection using V = 1/2 × ab^2^ method [length (a) and width (b) length of the tumor]. At 27 d after the injection, the mice were sacrificed and the subcutaneous tumor was weighed. This study was performed with approval from the Institutional Animal Care and Use Committee of Guizhou Medical University.

### Statistical analysis

The statistical analysis was conducted using SPSS 21.0 software (IBM, Somers, NY, USA). The correlation between miR-361-5p and circPOSTN or TPX2 was analyzed with Pearson’s correlation analysis. All data were exhibited as mean ± standard deviation, and *P* value less than 0.05 meant significant difference. The comparisons between two groups or among multiple groups were analyzed with Student’s *t*-test or one-way analysis of variance, respectively.

## Results

### CircPOSTN was overexpressed in glioma tissues and cells

**Fig. 1:**
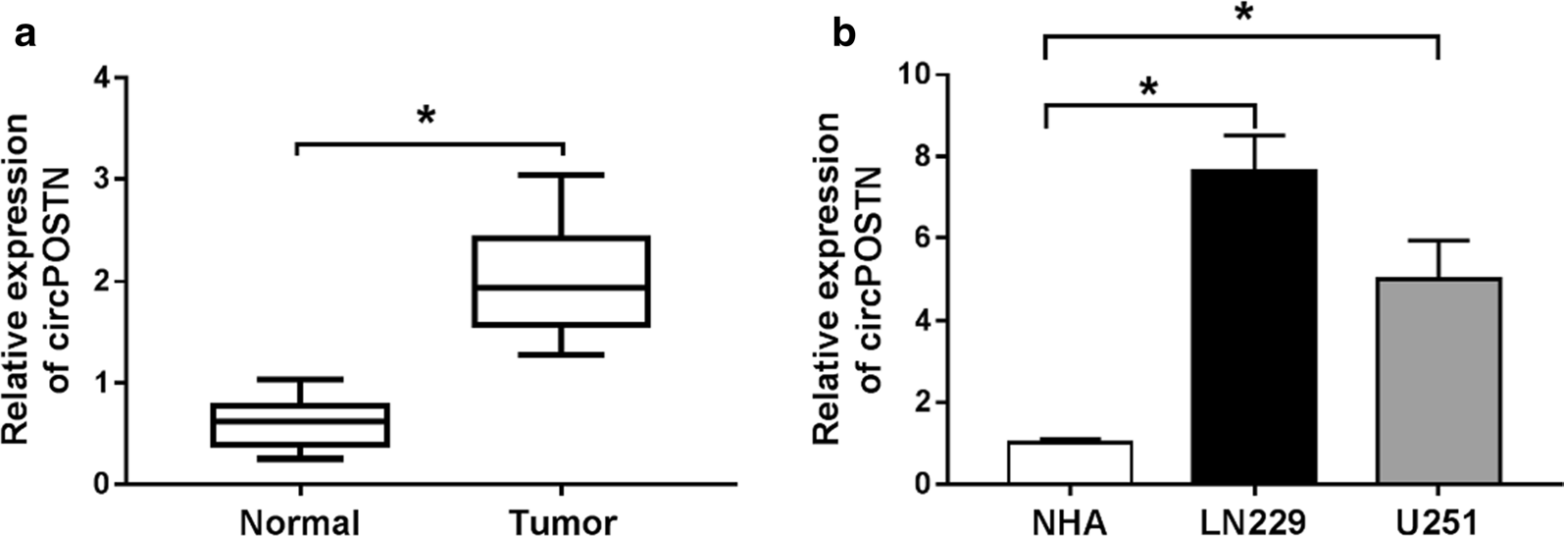
The expression level of circPOSTN in glioma tissues and cells. **a**, **b** The relative expression level of circPOSTN was determined with RT-qPCR assay in glioma tissues and normal tissues, as well as in NHA, LN229 and U251 cells (with GAPDH as housekeeping gene). *P < 0.05

**Fig. 2:**
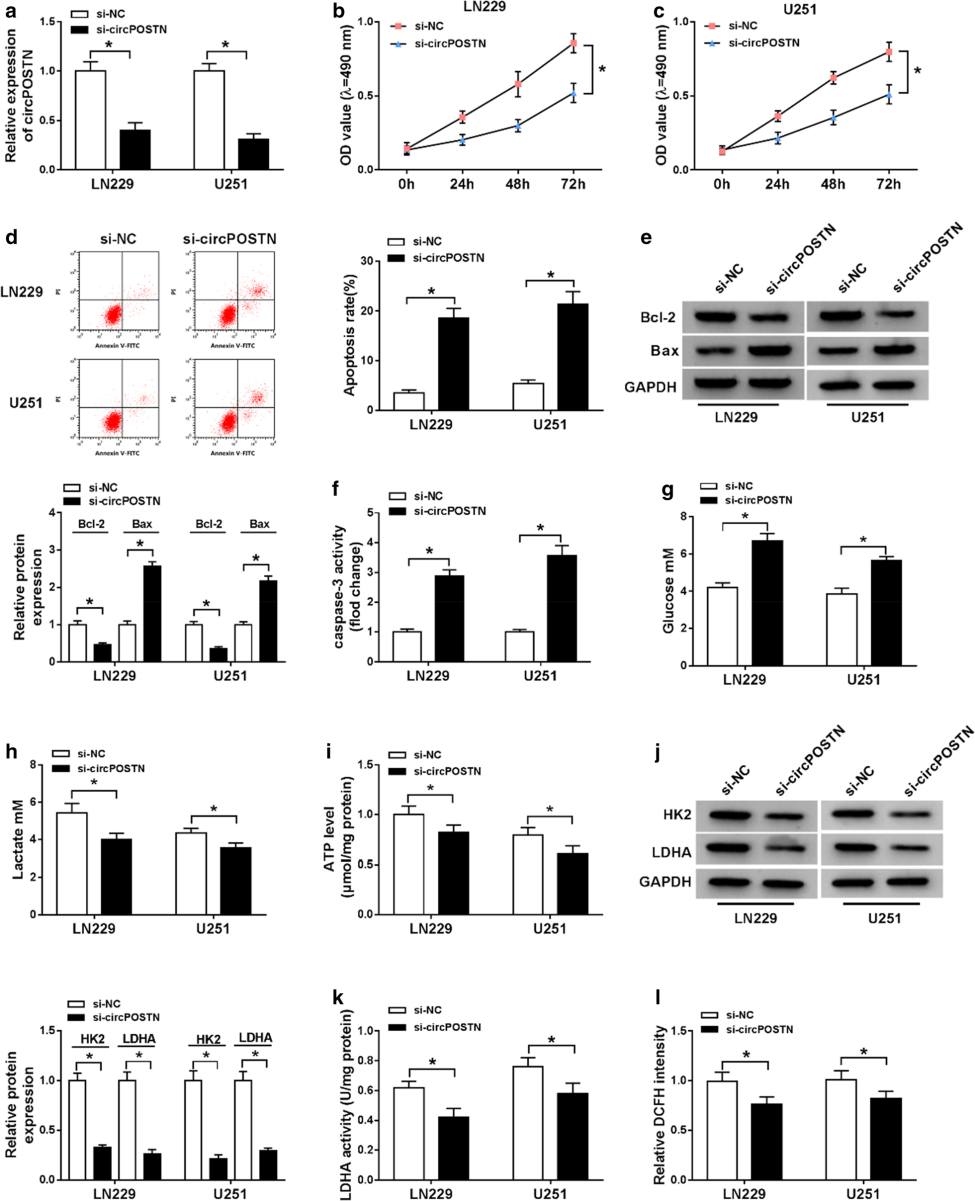
The influences of circPOSTN silencing on proliferation, apoptosis and aerobic glycolysis of glioma cells. **a**–**l** LN229 and U251 cells were transfected with si-circPOSTN or si-NC. **a** The interference efficiency of si-circPOSTN was analyzed with RT-qPCR assay in LN229 and U251 cells. **b**, **c** Effect of circPOSTN silencing on the cell viability of LN229 and U251 cells was assessed with MTT assay. **d** The apoptosis rate was computed with flow cytometry assay in transfected LN229 and U251 cells. **e** The western blot assay showed the expression levels of Bcl-2 and Bax in LN229 and U251 cells. **f** The caspase-3 activity was measured with a caspase-3 assay kit. **g**–**i** The concentration of glucose and lactate in the culture medium, as well as ATP production level were measured with a series of kits, respectively. **j** The protein expression levels of HK2 and LDHA were determined with western blot assay in transfected LN229 and U251 cells. **k**–**l** LDHA enzyme activity and ROS accumulation were evaluated in LN229 and U251 cells post-transfection with lactate dehydrogenase activity detection kit and reactive oxygen species assay kit, respectively. *P < 0.05

The RT-qPCR assay was implemented to figure out the expression level of circPOSTN in glioma tissues and normal tissues. As shown in Fig. 1a, results indicated that circPOSTN was drastically increased in glioma tissue samples compared with normal tissues. The expression level of circPOSTN was also assessed in glioma cells by RT-qPCR assay. Similarly, LN229 and U251 cells showed higher expression level of circPOSTN than NHA cells (Fig. 2e). Overall, above data concluded that circPOSTN was upregulated in glioma tissue and cells.

### CircPOSTN silencing impeded proliferation and aerobic glycolysis while induced apoptosis in glioma cells

As above results had suggested that circPOSTN was upregulated in glioma tissue and cells, and circPOSTN was knocked down in LN229 and U251 cells to analyze the role of circPOSTN. As presented in Fig. 2a, transfection of si-circPOSTN into LN229 and U251 cells obviously declined the level of circPOSTN. In addition, cell activity was dramatically downregulated in LN229 and U251 cells transfected with si-circPOSTN compared with that in cells transfected with si-NC (Fig. 2b, c). We also found that downregulation of circPOSTN led to cell apoptosis in LN229 and U251 cells (Fig. 2d). Further study of western blot assay also showed that Bcl-2 was decreased and Bax was increased in LN229 and U251 cells after silencing of circPOSTN (Fig. 2e). Moreover, through the caspase-3 assay kit assay in LN229 and U251 cells, the obvious promotion of caspase-3 activity (nearly threefold) was found in LN229 and U251 transfected with si-circPOSTN compared with control group (Fig. 2f). To assess the role of circPOSTN in aerobic glycolysis of LN229 and U251 cells, a series of assay kits were used to measure concentration of glucose and lactate in the medium, as well as cellular ATP level. As shown in Fig. 2g–i, the results revealed that knockdown of circPOSTN resulted in the decrease of glucose consumption, lactate production, and ATP level. Considering HK2 and LDHA were key enzymes for glucose metabolism, HK2 and LDHA were quantified with western blot assay. The downregulation of circPOSTN prominently inhibited HK2 and LDHA expression in LN229 and U251 cells (Fig. 2j). Furthermore, enzyme activity of LDHA was decreased in LN229 and U251 cells after treatment with si-circPOSTN (Fig. 2k). Knockdown of circPOSTN could decline ROS accumulation in LN229 and U251 cells (Fig. 2l). In summary, circPOSTN played an important function in proliferation, aerobic glycolysis, and apoptosis of glioma cells.

### CircPOSTN directly targeted miR-361-5p in glioma cells

**Fig. 3:**
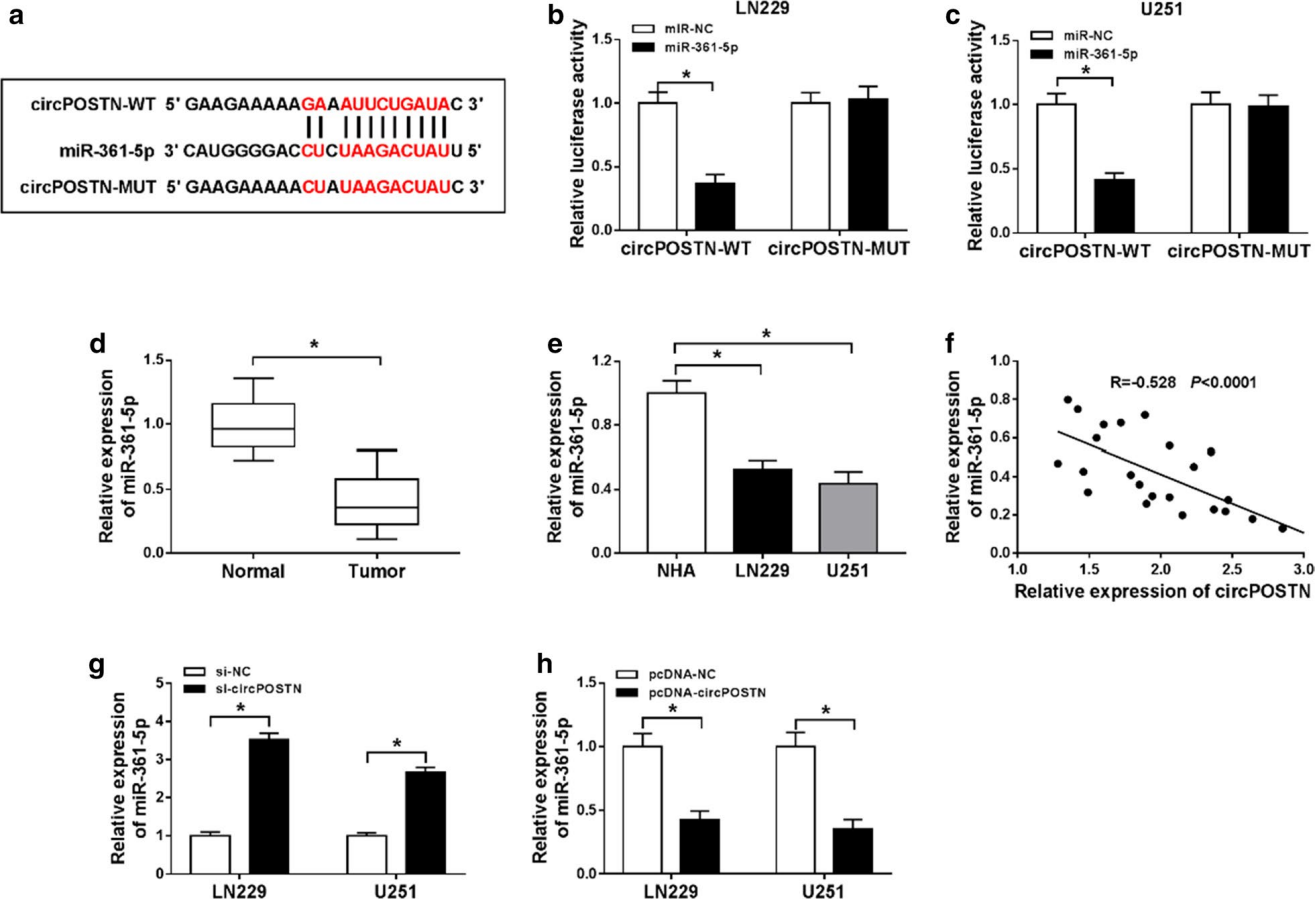
MiR-361-5p was a direct target of circPOSTN in glioma cells. **a** Binding region between miR-361-5p and circPOSTN was predicted by StarBase3.0. **b**, **c** The relationship between miR-361-5p and circPOSTN was confirmed by dual-luciferase reporter assay. **d**, **e** The relative expression level of miR-361-5p was quantified using RT-qPCR assay in glioma tissues and brain tissues, as well as in NHA, LN229 and U251 cells. **f** The correlation analysis between miR-361-5p and circPOSTN was analyzed by Pearson’s correlation analysis in glioma tissues. **g**, **h** The RT-qPCR assay was executed to observe the expression level of miR-361-5p in LN229 and U251 cells introduced with si-NC, si-circPOSTN, pcDNA-NC, or pcDNA-circPOSTN. *P < 0.05

To determine the involvement of target miRNA of circPOSTN in glioma cells, StarBase3.0 was used to show putative binding sites between circPOSTN and miR-361-5p (Fig. 3a). TAfter that, we performed dual-luciferase reporter assay, and the results revealed that the luciferase activity of the circPOSTN-WT reporter was declined in LN229 and U251 cells introduced with miR-361-5p compared with control group, while introduction with miR-361-5p did not change luciferase activity of the circPOSTN-MUT reporter in LN229 and U251 cells compared with control group (Fig. 3b, c). Moreover, as indicated in Fig. 3d, e, miR-361-5p level was lower in glioma tissues and cells than that in normal tissues and NHA cells, respectively. Additionally, Pearson’s correlation analysis showed that circPOSTN negatively correlated with miR-361-5p in glioma tissues (Fig. 3f). Furthermore, miR-361-5p was increased in LN229 and U251 cells introduced with si-circPOSTN compared with control, conversely, overexpression of circPOSTN triggered opposite result (Fig. 3g, h). Collectively, circPOSTN negatively regulated miR-361-5p expression in glioma cells.

### CircPOSTN impeded proliferation and induced apoptosis in glioma cells by targeting miR-361-5p

**Fig. 4:**
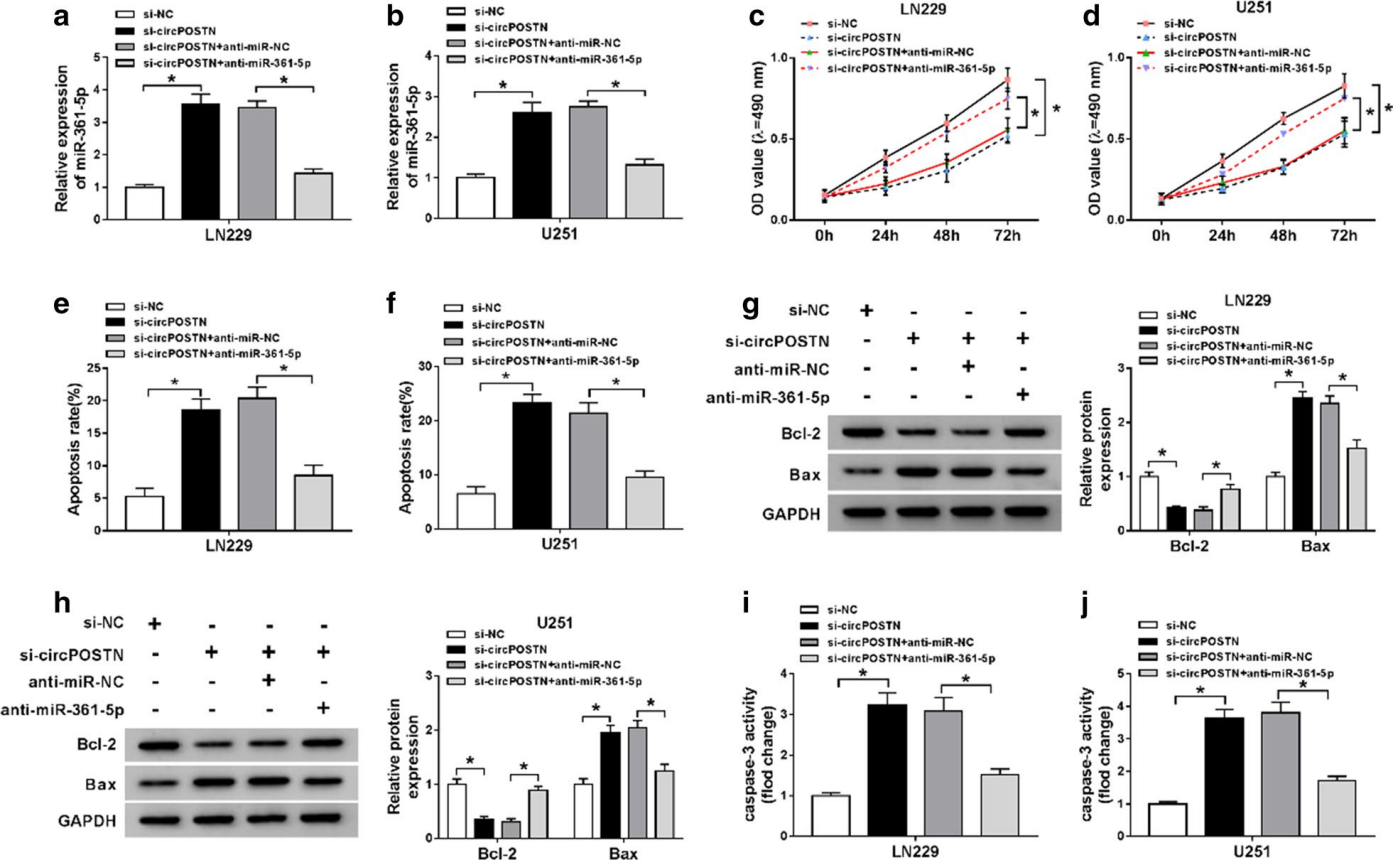
Knockdown of circPOSTN mediated-effects on proliferation and apoptosis of glioma cells could be eliminated by silencing miR-361-5p. **a**–**j** LN229 and U251 cells were transfected with si-NC, si-circPOSTN, si-circPOSTN + anti-miR-NC, or si-circPOSTN + anti-miR-361-5p. **a**, **b** The relativity expression level of miR-361-5p was analyzed with RT-qPCR assay in LN229 and U251 cells. **c**, **d** MTT assay was administrated to assess cell viability of LN229 and U251 cells after transfection. **e**, **f** The apoptosis of transfected LN229 and U251 cells was monitored by flow cytometry. **g**, **h** The western blot assay was employed to show the expression levels of Bcl-2 and Bax in LN229 and U251 cells. **i**, **j** The caspase-3 activity was examined by caspase-3 assay kit. *P < 0.05

Given circPOSTN negatively regulated miR-361-5p expression, we further explored the relationship of circPOSTN and miR-361-5p on the proliferation and apoptosis of glioma cells. The results of RT-qPCR assay demonstrated that knockdown of circPOSTN aggrandized miR-361-5p expression in LN229 and U251 cells, which could be counteracted by transfection with miR-361-5p inhibitor (Fig. 4a, b). Moreover, miR-361-5p inhibitor rescued the inhibitory effect of circPOSTN silencing on the proliferation of glioma cells (Fig. 4c, d). Knockdown of miR-361-5p relieved the enhancement effect on apoptosis of glioma cells induced by silencing circPOSTN (Fig. 4e, f). Consistently, Bcl-2 was declined and Bax was increased in LN229 and U251 cells after knockdown circPOSTN, which were abolished by miR-361-5p inhibitor (Fig. 4g, h). At the meanwhile, activity of caspase-3 was reinforced in circPOSTN silencing-cells, which was inverted by miR-361-5p knockdown (Fig. 4i, j). Taken together, these results revealed that knockdown of circPOSTN exerted the tumor-suppressive effects by targeting miR-361-5p.

### Effects of circPOSTN silencing on aerobic glycolysis of glioma cells were abrogated by miR-361-5p knockdown

**Fig. 5:**
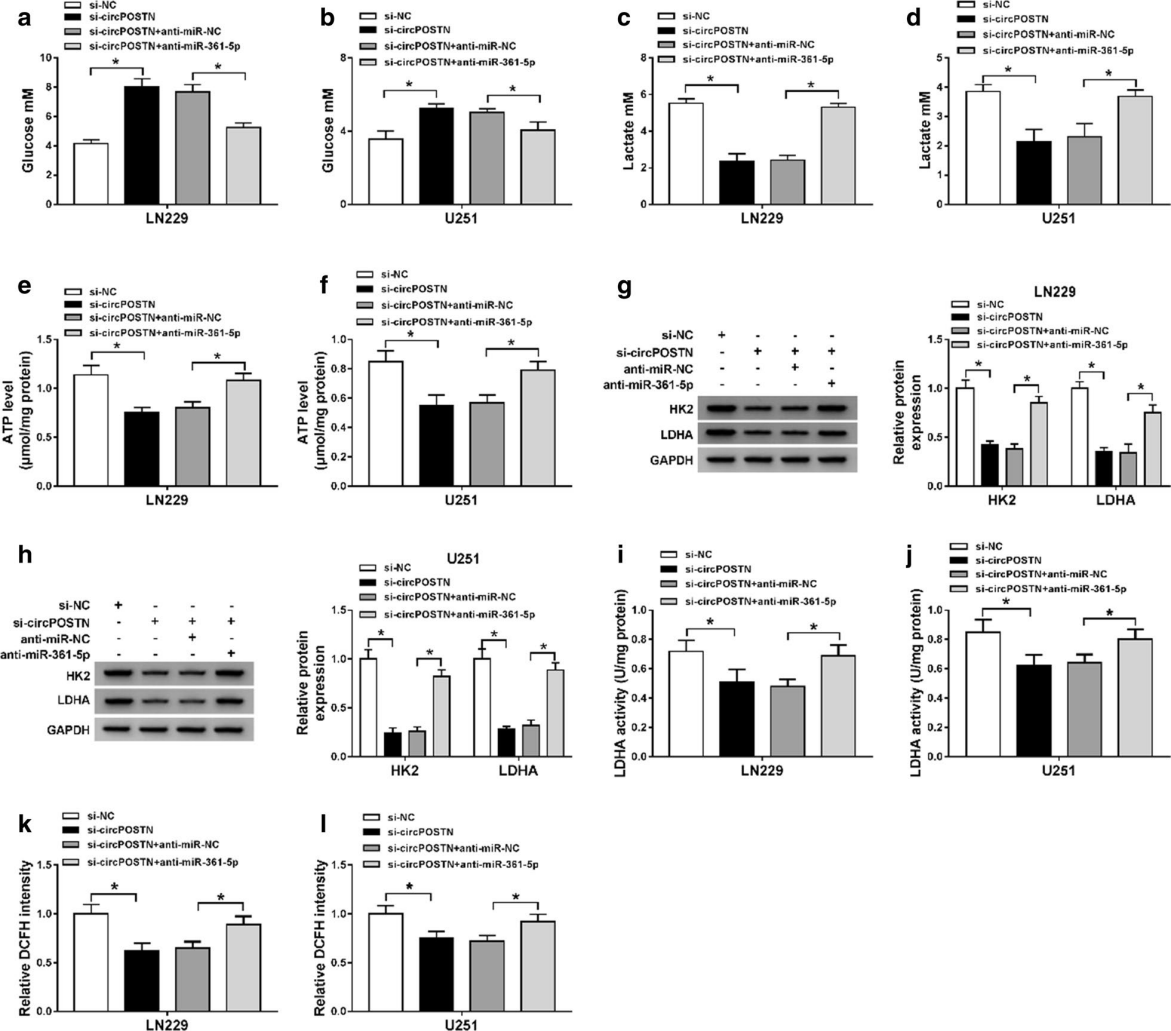
CircPOSTN silencing inhibited aerobic glycolysis of glioma cells via regulating miR-361-5p. **a**–**l** LN229 and U251 cells were transfected with si-NC, si-circPOSTN, si-circPOSTN + anti-miR-NC, or si-circPOSTN + anti-miR-361-5p. **a**–**f** The concentration of glucose and lactate, as well as cellular ATP level were detected with different kits. **g**, **h** The protein expression levels of HK2 and LDHA in LN229 and U251 cells were measured with western blot assay. **i**–**l** The enzyme activity of LDHA and ROS level were measured in transfected LN229 and U251 cells. *P < 0.05

To further confirm the interaction relationship between circPOSTN and miR-361-5p, glioma cells were transfected with si-NC, si-circPOSTN, si-circPOSTN + anti-miR-NC, or si-circPOSTN + anti-miR-361-5p. Downregulation of circPOSTN led to decrease of lactate production and glucose consumption in the culture medium, and intracellular ATP, revealing glucose metabolism was declined, which were prominently reversed by miR-361-5p knockdown (Fig. 5a–f). We also measured protein expression levels of key glycolytic enzymes HK2 and LDHA in glioma cells, as well as enzyme activity of LDHA. As presented in Fig. 5g–j, when compared with si-NC group, the levels of HK2 and LDHA, as well as enzyme activity of LDHA were obviously decreased in si-circPOSTN group, whereas knockdown of miR-361-5p eliminated the reduction effects. In addition, knockdown of miR-361-5p also abolished inhibition effect on ROS accumulation of glioma cells caused by silencing circPOSTN (Fig. 5k, l). The data suggested that circPOSTN/miR-361-5p axis regulated aerobic glycolysis of glioma cells.

### CircPOSTN sponged miR-361-5p to regulate TPX2 expression in glioma cells

**Fig. 6:**
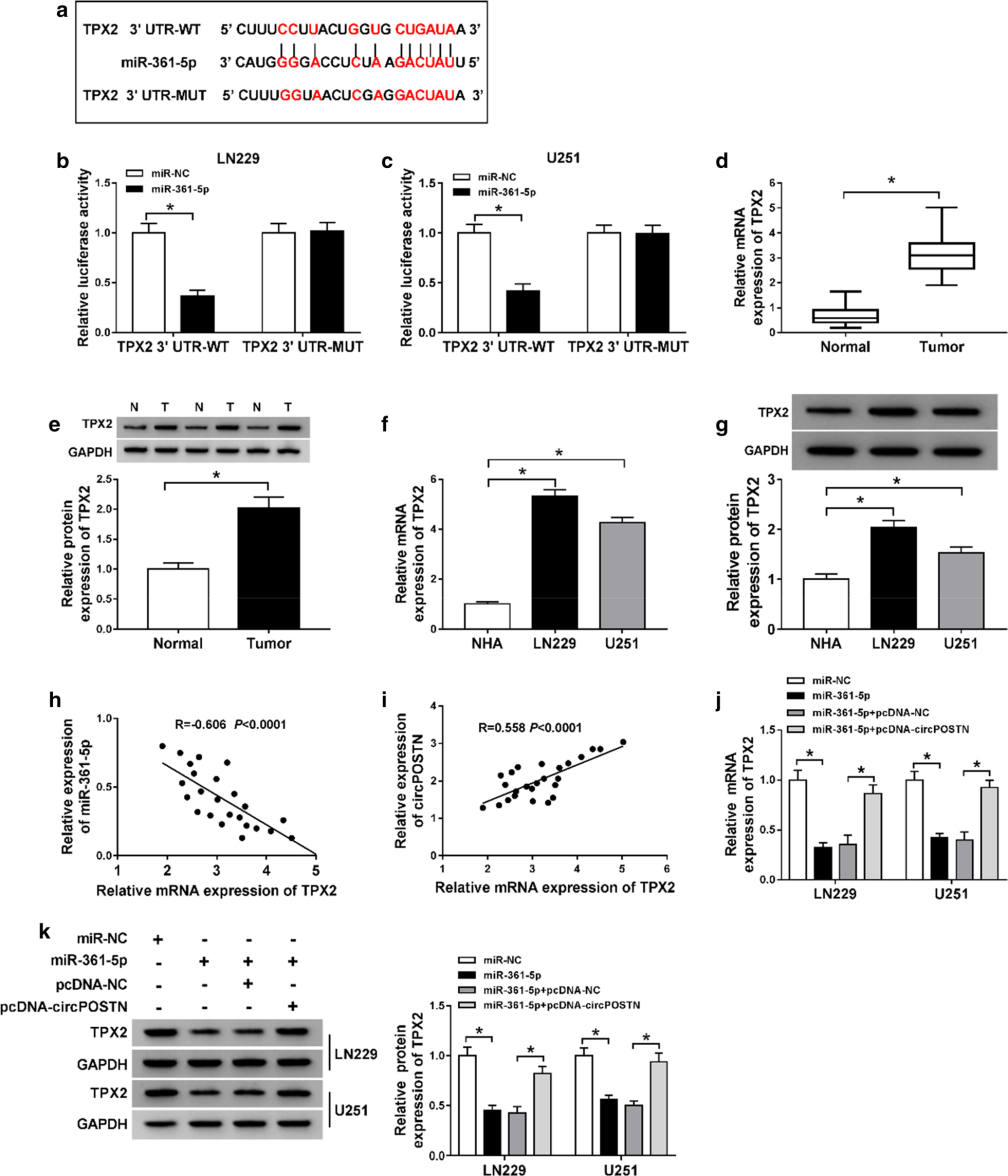
CircPOSTN regulated TPX2 expression via sponging miR-361-5p in glioma cells. **a** The binding sequences between miR-361-5p and TPX2, and matched mutant sites were shown. **b**, **c** Luciferase activity was determined by dual-luciferase reporter assay in LN229 and U251 cells following co-transfection with TPX2 3′UTR-WT or TPX2 3′UTR-MUT and miR-361-5p or miR-NC according to the design. **d**–**g** The mRNA and protein expression levels of TPX2 were estimated using RT-qPCR and western blot assays in glioma tissues and cells, along with controls. **h**, **i** The correlation analysis between TPX2 and miR-361-5p or circPOSTN was conducted by Pearson’s correlation analysis. **j**, **k** The RT-qPCR and western blot assays were recruited to evaluate the expression levels of TPX2 level in LN229 and U251 cells transfected with miR-NC, miR-361-5p, miR-361-5p + pcDNA-NC or miR-361-5p + pcDNA-circPOSTN. *P < 0.05

Analogously, StarBase3.0 was applied to predict the possible target mRNA of miR-361-5p. As displayed in Fig. 6a, TPX2 contained a binding site of miR-361-5p. The data of dual-luciferase reporter assay suggested that miR-361-5p overexpression remarkably declined the luciferase activity of the wild-type reporter vector (TPX2 3′UTR-WT), but not the mutant reporter vector (TPX2 3′UTR-MUT) in LN229 and U251 cells (Fig. 6b, c). Moreover, the results of RT-qPCR and western blot assays implied that TPX2 was highly expressed in glioma tissues and cells with respect to matched controls (Fig. 6d–g). Furthermore, a negative association between miR-361-5p and TPX2 was observed in Fig. 6h. Moreover, Pearson’s correlation analysis suggested that TPX2 was positively correlated with circPOSTN expression in glioma tissues (Fig. 6i). The miR-361-5p overexpression distinctly downregulated the expression level of TPX2, which was eliminated by circPOSTN overexpression (Fig. 6j, k). All these data confirmed that circPOSTN may act as a miR-361-5p sponge in glioma cells.

### Knockdown of TPX2 impeded proliferation and aerobic glycolysis while increased apoptosis in glioma cells

**Fig. 7:**
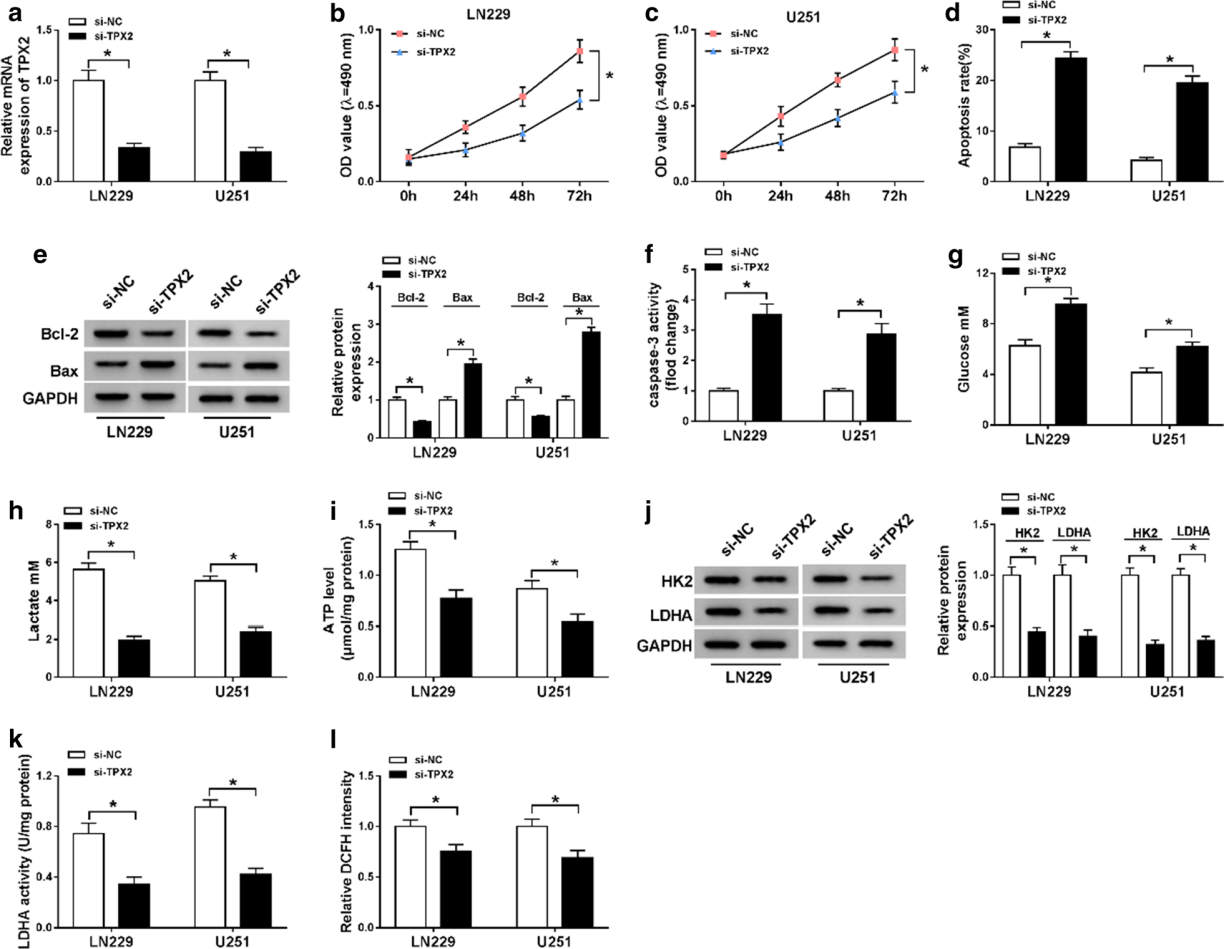
TPX2 regulated proliferation, apoptosis, and aerobic glycolysis in glioma cells. **a**–**l** LN229 and U251 cells were introduced with si-NC or si-TPX2. **a** The transfection efficiency of si-TPX2 was checked with RT-qPCR assay in LN229 and U251 cells. **b**, **c** The cell viability of LN229 and U251 cells was determined with MTT assay. **d** The apoptosis rate of transfected LN229 and U251 cells was represented by flow cytometry assay. **e** The western blot assay was used to assay the expression levels of Bcl-2 and Bax in LN229 and U251 cells. **f** The activity of caspase-3 was detected with a caspase-3 assay kit. **g**–**i** The glucose, lactate, and ATP production levels were shown. **j** The protein expression levels of HK2 and LDHA were estimated by western blot assay in LN229 and U251 cells. **k**, **l** LDHA enzyme activity and ROS content were evaluated in LN229 and U251 cells post-transfection. *P < 0.05

To further figure out the role of TPX2 in glioma, we knocked down TPX2 in LN229 and U251 cells by introducing with si-TPX2. As shown in Fig. 7a, the expression level of TPX2 was significantly lower in si-TPX2 group than that in si-NC group. Knockdown of TPX2 conspicuously dwindled cell proliferation ability in LN229 and U251 cells compared with the control (Fig. 7b, c). Conversely, apoptosis rate of LN229 and U251 cells was increased in si-TPX2 group in comparison with si-NC group (Fig. 7d). Expression of Bax and activity of caspase-3 were strengthened, while Bcl-2 was reduced in LN229 and U251 cells by TPX2 siRNA (Fig. 7e, f). In addition, aerobic glycolysis was inhibited in LN229 and U251 cells treatment with si-TPX2 by impeding glucose consumption and declining accumulation of lactate and ATP production (Fig. 7g–i). The results of western blot assay indicated that the expression levels of HK2 and LDHA were decreased in si-TPX2 group when compared with si-NC group (Fig. 7j), at the same time, enzyme activity of LDHA was inhibited by TPX2 silencing (Fig. 7k). Besides, fluorescence density of 2,7-Dichlorodi-hydrofluorescein diacetate was lower in si-TPX2 group than that in si-NC group (Fig. 7l), suggesting ROS was declined in si-TPX2 group. Thus, it was concluded that knockdown of TPX2 impeded process of glioma by regulating proliferation, apoptosis, and aerobic glycolysis in glioma cells.

### CircPOSTN regulated glucose metabolism in glioma cells

**Fig. 8:**
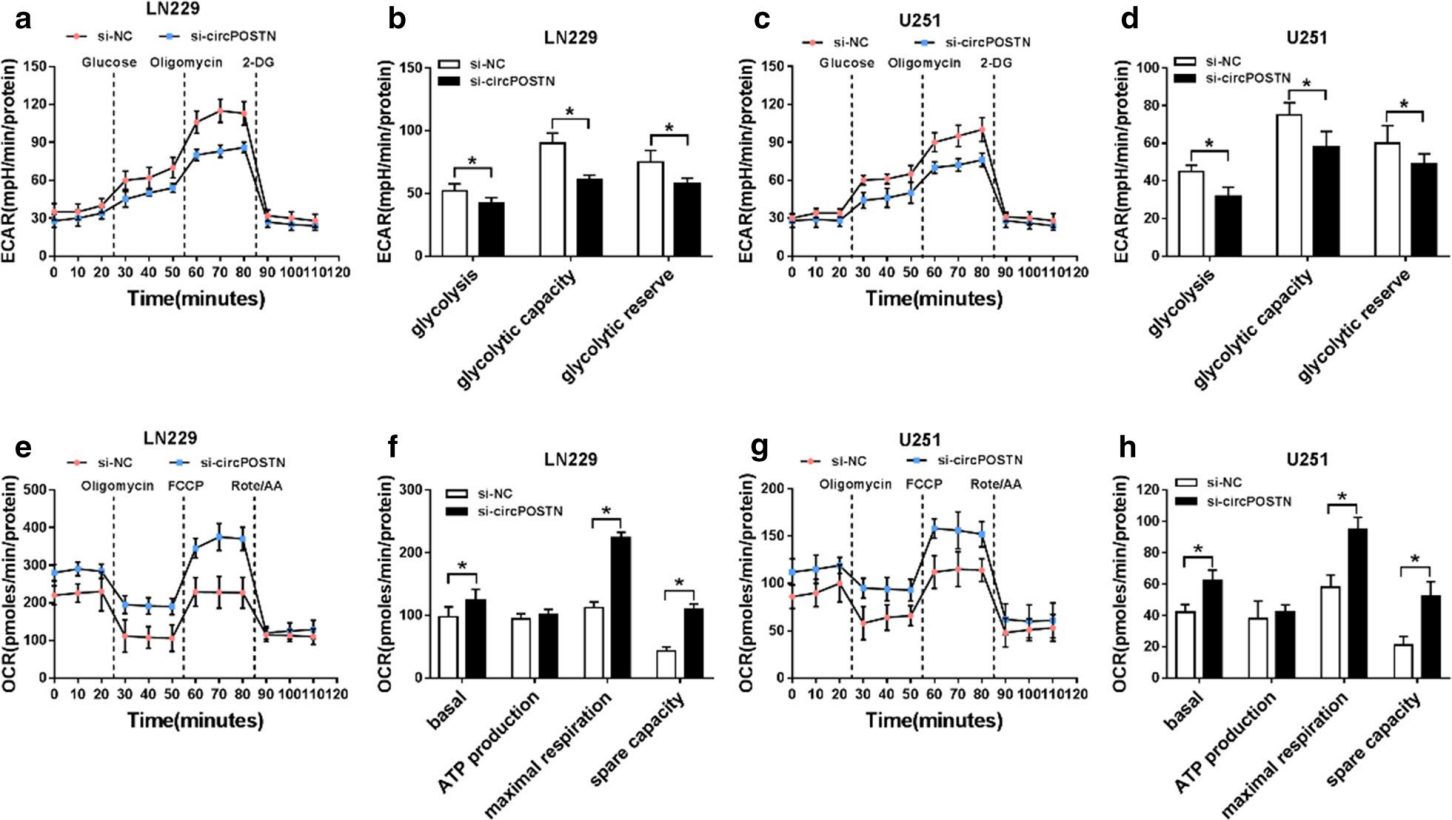
Extracellular acidification rate and oxygen consumption rate assays in glioma cells. **a**–**d** The extracellular acidification rate curve was presented by Seahorse XF assay in LN229 and U251 cells transfected with si-circPOSTN or si-NC. **e**–**h** The quantification of oxygen consumption rate was measured with Seahorse XF assay in LN229 and U251 cells with circPOSTN knockdown. *P < 0.05

Based on above results, Seahorse XF assay was used to further evaluate the effects of circPOSTN silencing on glucose metabolism in glioma cells. The results revealed that ECAR was declined after circPOSTN knockdown (Fig. 8a–d). In addition, a higher basal mitochondrial OCR and maximal OCR were presented in si-circPOSTN group that in si-NC group, as demonstrated by OCR analysis (Fig. 8e–h). Collectively, above results implied that circPOSTN enhanced glioma cells’ metabolic rewiring in vitro.

### CircPOSTN knockdown suppressed tumor growth in nude mice

**Fig. 9:**
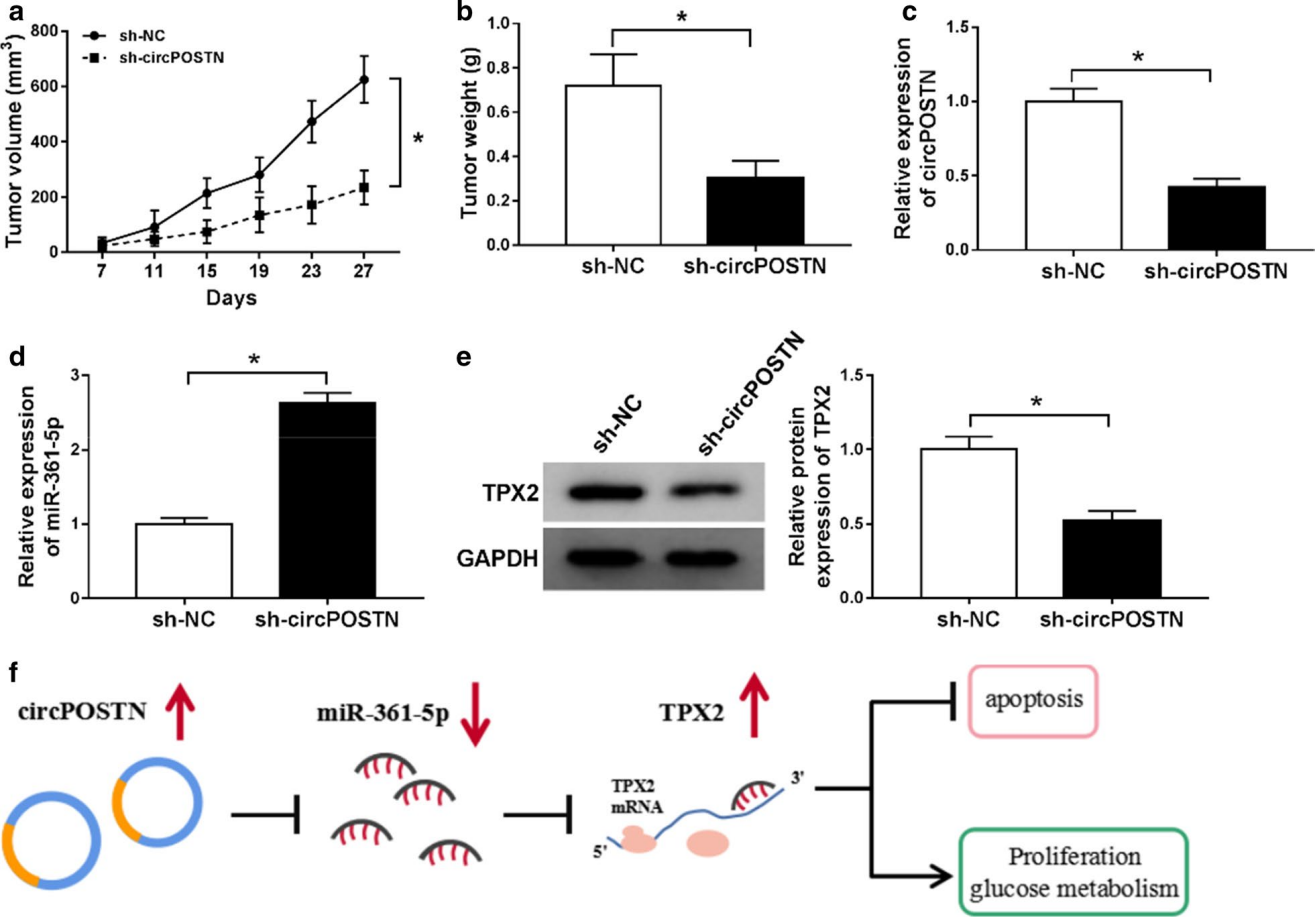
Silencing of circPOSTN repressed glioma tumor growth in vivo. **a**, **b** Tumor volume and weight were presented. **c**, **d** The expression levels of circPOSTN and miR-361-5p in dissected tumor tissues were estimated with RT-qPCR assay. **e** Western blot assay was executed to test protein level of TPX2 in dissected tumor tissues. **f** The diagrammatic representation of our conclusion was shown. *P < 0.05

The in vivo tumor model was used to certify the above findings. The results indicated that the formed tumors from the circPOSTN knockdown LN229 cells exhibited slower growth rate than that in control group, consistently, sh-circPOSTN group had smaller tumors weight compared with sh-NC group (Fig. 9a, ). In addition, circPOSTN was declined and miR-361-5p was increased in sh-circPOSTN group in comparison with sh-NC group (Fig. 9c, d). The results of western blot assay suggested that circPOSTN knockdown impeded TPX2 expression (Fig. 9e). In addition, the diagrammatic representation of our results suggested that circPOSTN/miR-361-5p/TPX2 axis regulated cell growth, apoptosis and aerobic glycolysis in glioma cells. These results implied that circPOSTN silencing impeded glioma tumor growth in vivo by regulation of miR-361-5p and TPX2 in part.

## Discussion

The previous studies had indicated that circPOSTN was overexpressed in glioma tissues compared with normal tissues [4, 19]. Consistently, our results also confirmed that circPOSTN was increased in glioma tissues and cells compared with corresponding control groups. In addition, mechanistic analysis experiments revealed that circPOSTN silencing impeded glioma cells growth, while induced apoptosis by targeting miR-361-5p/TPX2 axis.

The aerobic glycolysis is a favored way for cancer cells to obtain energy and several glycolytic intermediates, whereas biological macromolecules are required for tumor cells growth and proliferation [20]. HK2, a key metabolic enzyme, plays crucial role in glucose uptake and the Warburg effect [21]. For example, Lan et al. reported that miR-448 functioned as a tumor-inhibiting factor by interfering with HK2 and LDHA that are regulators in glycolysis [22]. Analogously, HK2 was upregulated in glioblastoma multiforme tumors, resulting in enhancement effects on proliferation and therapeutic resistance of tumor cells [23]. Consequently, in current study, we monitored HK2 and LDHA expression, as well as LDHA enzyme activity to analyze aerobic glycolysis of glioma cells. Furthermore, the Glycolysis Stress Test assay was applied to test the effects of circPOSTN silencing on glycolysis. Our results confirmed that knockdown of circPOSTN constrained aerobic glycolysis of glioma cells. We also observed that silencing of circPOSTN could inhibit HK2 and LDHA expression in glioma cells. More importantly, our data indicated that the involvement of circPOSTN in the pathogenesis of glioma might be through affecting aerobic glycolysis and cell proliferation.

Several studies have found that miR-361-5p exerted its role by acting as a carcinoma inhibitor in human cancers, including squamous cell carcinoma of the skin and prostate cancer [24, 25]. Our findings indicated that miR-361-5p was downregulated in glioma, and it was reversely regulated by circPOSTN. Liu et al. revealed that miR-361-5p targeted secondary wall-associated NAC domain protein 1 to decline MMP-2 transcription, which impeded glioma migration and invasion [26]. Given miRNAs are pivotal regulators for post-transcriptional gene expression [27, 28]. Currently, bioinformatics database and dual-luciferase reporter assay verified that miR-361-5p interacted with circPOSTN by a certain binding site, thereby knockdown of miR-361-5p mitigated the effects of circPOSTN silencing on apoptosis, proliferation and aerobic glycolysis in glioma cells.

Next, we further explored the possible downstream target of miR-361-5p/circPOSTN. Currently, glioma tissues and cells exhibited upregulation expression of TPX2, and TPX2 was inversely correlated with miR-361-5p expression in glioma tumor tissues. Mechanistically, Chen et al. revealed that miR-1294 could mediate TPX2 expression to regulate proliferation and chemosensitivity in glioma cells [29]. In this study, we found that miR-361-5p could interact with TPX2 in glioma cells by dual-luciferase reporter assay. Furthermore, downregulation of TPX2 contributed to apoptosis and inhibited proliferation and aerobic glycolysis in glioma cells. Not surprisingly, Li et al. also pointed out that silencing of TPX2 blocked cell growth and enhanced apoptosis in glioma cells [30].

In summary, we verified miR-361-5p as a direct functional target of circPOSTN by dual-luciferase assay. Mechanistically, circPOSTN serves as a competing endogenous RNA in glioma by sponging miR-361-5p. Most importantly, the current study was the first to show that the detailed role and mechanism of circPOSTN/miR-361-5p/TPX2 axis in aerobic glycolysis of glioma cells, providing a novel and potential therapy strategy for glioma (Table 1).

**Table 1:** The sequences of primers were listed

Gene	Sense	Antisense
circPOSTN	5′-AAGCGCTTTAGCACCTTCCT-3′	5′-CTTCCTCACGGGTGTGTCTC-3′
miR-361-5p	5′-GCCGAGTTATCAGAATCTCCA-3′	5′-CTCAACTGGTGTCGTGGA-3′
TPX2	5′-AACCACCCACCGAGCCTAT-3′	5′-CACCCACAACATCTTCCAAAA-3′
GAPDH	5′-TCCCATCACCATCTTCCAGG-3′	5′-GATGACCCTTTTGGCTCCC-3′
U6	5′-AACGCTTCACGAATTTGCGT-3′	5′-CTCGCTTCGGCAGCACA-3′
si-circPOSTN	5′-ACUAAUUUCAUUCAAUUUCCU-3′	5′-GAAAUUGAAUGAAAUUAGUUG-3′
si-TPX2	5′-UCAAGAACCGUUAUUAGCCGA-3′	5′-GGCUAAUAACGGUUCUUGAUA-3′
si-NC	5′-GGCCUAAAGUAGUAGCUAUTT-3′	5′-AUAGCUACUACU UUAGGCCTT-3′

## Conclusion

In conclusion, we confirmed that circPOSTN played a tumorigenic role in glioma. Moreover, deletion of circPOSTN or TPX2 significantly repressed cell proliferation and aerobic glycolysis, while induced apoptosis of glioma cells. Additionally, miR-361-5p, interacted with TPX2, was a target gene circPOSTN, suggesting circPOSTN/miR-361-5p/TPX2 axis may be a new therapeutic target for the diagnosis and treatment of glioma in the future (Additional files 1, 2, 3, 4, 5, 6, 7, 8, 9, 10).

## Supplementary information

**Additional file 1.** Predicted other target miRNAs of circPOSTN.

**Additional file 2.** The expression level of circPOSTN in glioma tissues and cells.

**Additional file 3.** The influences of circPOSTN silencing on proliferation, apoptosis and aerobic glycolysis of glioma cells.

**Additional file 4.** MiR-361-5p was a direct target of circPOSTN in glioma cells.

**Additional file 5.** Knockdown of circPOSTN mediated-effects on proliferation and apoptosis of glioma cells could be eliminated by silencing miR-361-5p.

**Additional file 6.** CircPOSTN silencing inhibited aerobic glycolysis of glioma cells via regulating miR-361-5p.

**Additional file 7.** CircPOSTN regulated TPX2 expression via sponging miR-361-5p in glioma cells.

**Additional file 8.** TPX2 regulated proliferation, apoptosis, and aerobic glycolysis in glioma cells.

**Additional file 9.** Extracellular acidification rate and oxygen consumption rate assays in glioma cells.

**Additional file 10.** Silencing of circPOSTN repressed glioma tumor growth in vivo.

## Data Availability

The analyzed data sets generated during the present study are available from the corresponding author on reasonable request.

## Abbreviations

circRNA: Circular RNA
TPX2: Xenopus kinesin-like protein 2
ROS: Reactive oxygen species
GBM: Glioblastoma multiforme
NHA: Normal human astrocytes
LN229: Glioma cell line
RT-qPCR: Real-time quantitative polymerase chain reaction
PI: Propidium iodide
RIPA: The radio-immunoprecipitation assay
ECAR: Extracellular acidification rate
