# Supplementary figures and images for: CircPOSTN/miR-361-5p/TPX2 axis regulates cell growth, apoptosis and aerobic glycolysis in glioma cells
Source: Cancer Cell Int. 2020 Aug 6;20:374. doi: 10.1186/s12935-020-01454-x (PMC7409503; doi:10.1186/s12935-020-01454-x)

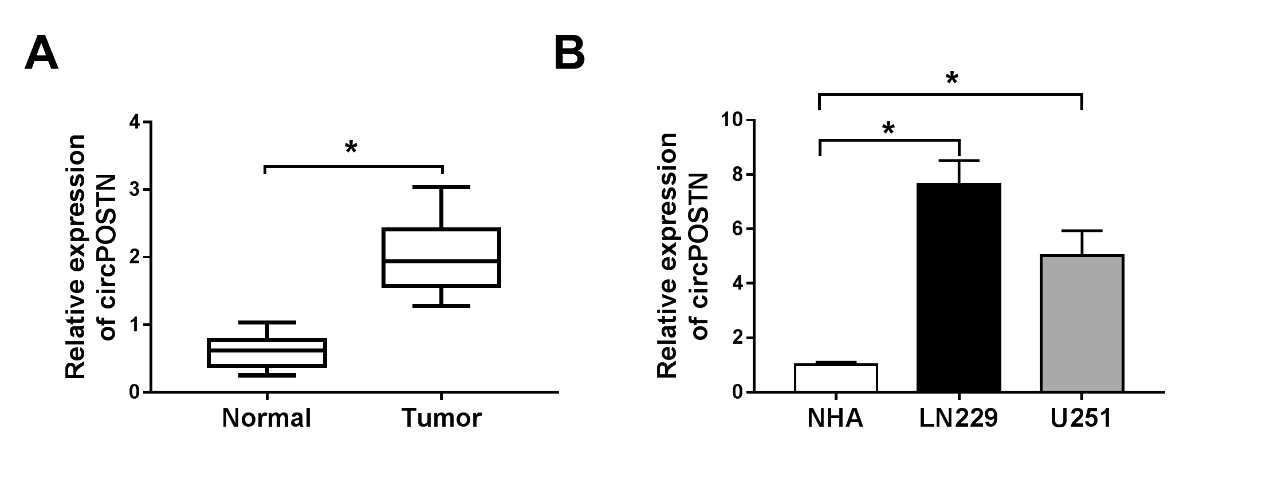

Supplement: Supplementary file 2 — Additional file 2. The expression level of circPOSTN in glioma tissues and cells. [file 12935_2020_1454_MOESM2_ESM.png]

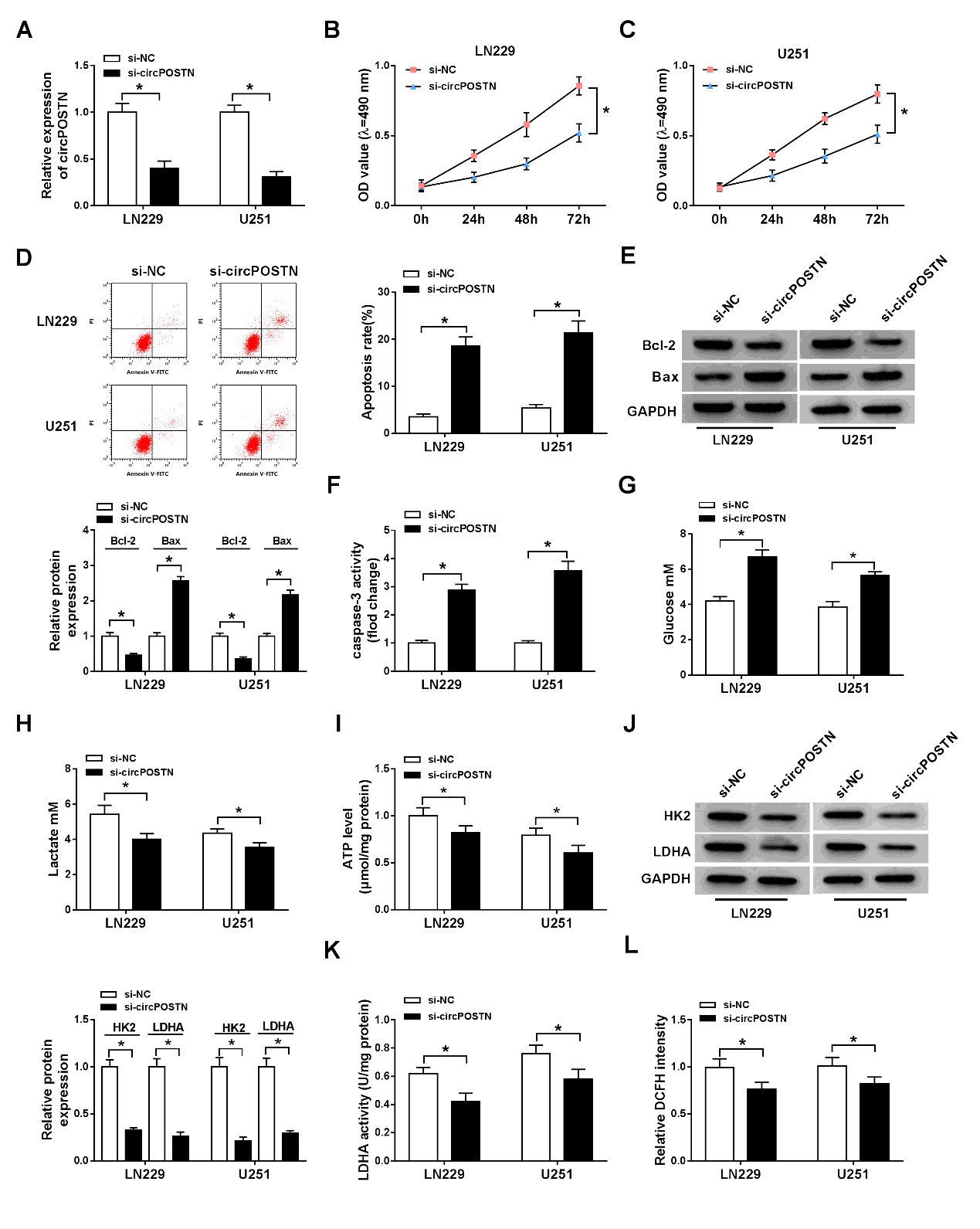

Supplement: Supplementary file 3 — Additional file 3. The influences of circPOSTN silencing on proliferation, apoptosis and aerobic glycolysis of glioma cells. [file 12935_2020_1454_MOESM3_ESM.png]

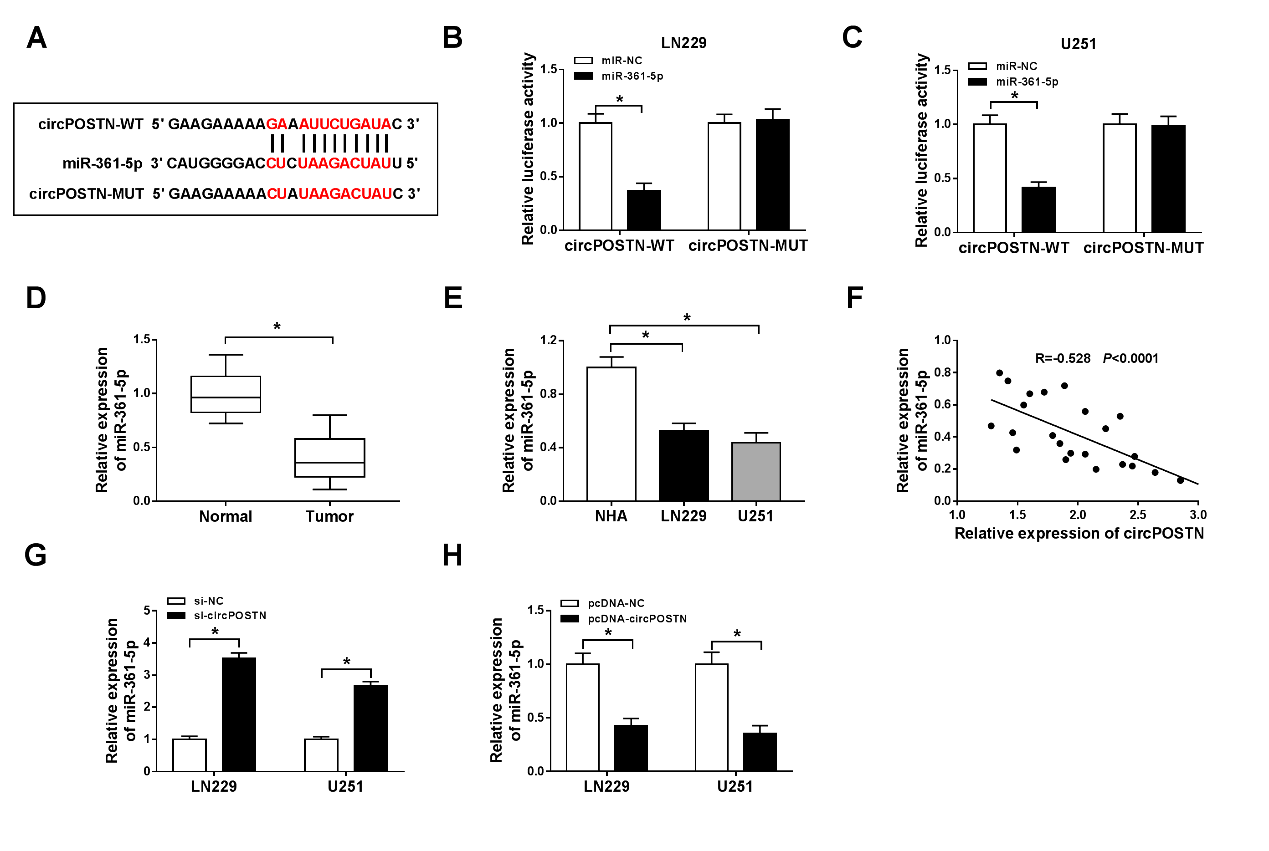

Supplement: Supplementary file 4 — Additional file 4. MiR-361-5p was a direct target of circPOSTN in glioma cells. [file 12935_2020_1454_MOESM4_ESM.png]

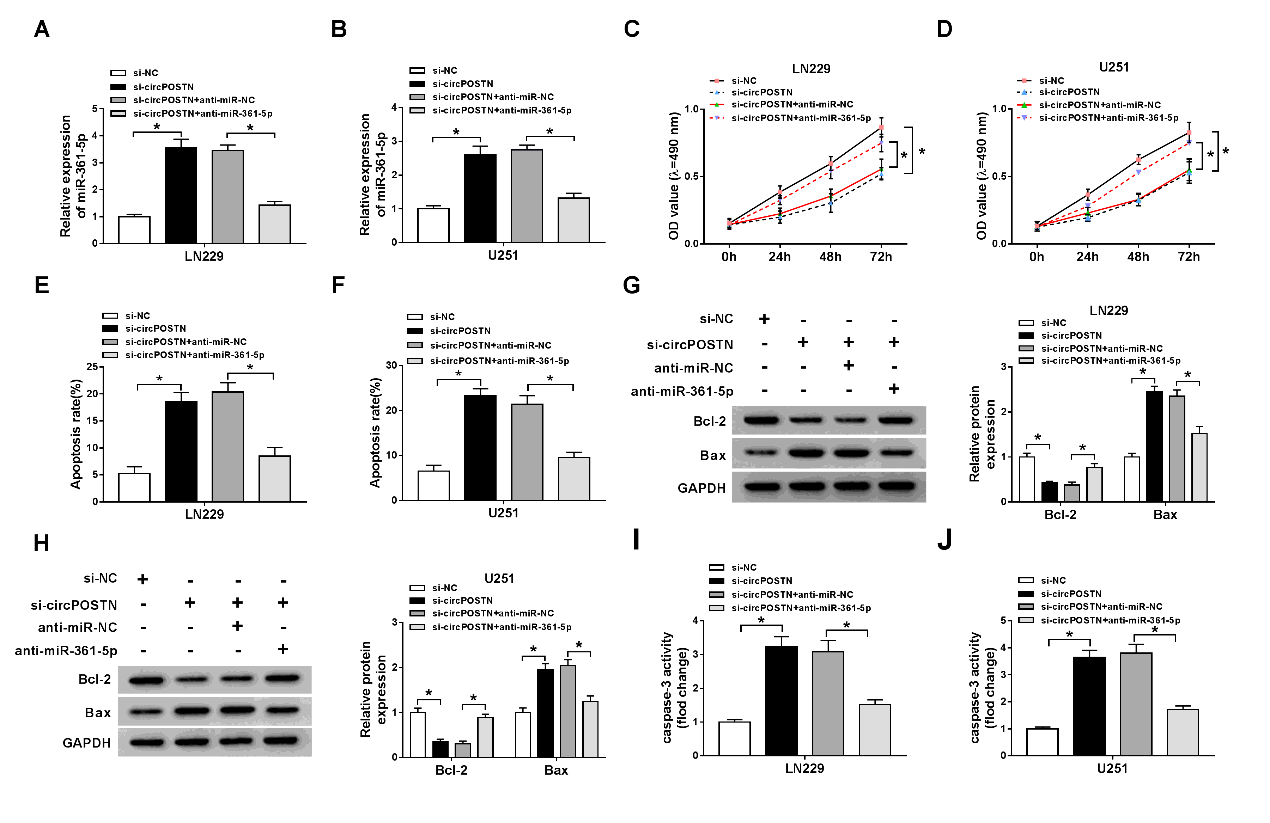

Supplement: Supplementary file 5 — Additional file 5. Knockdown of circPOSTN mediated-effects on proliferation and apoptosis of glioma cells could be eliminated by silencing miR-361-5p. [file 12935_2020_1454_MOESM5_ESM.png]

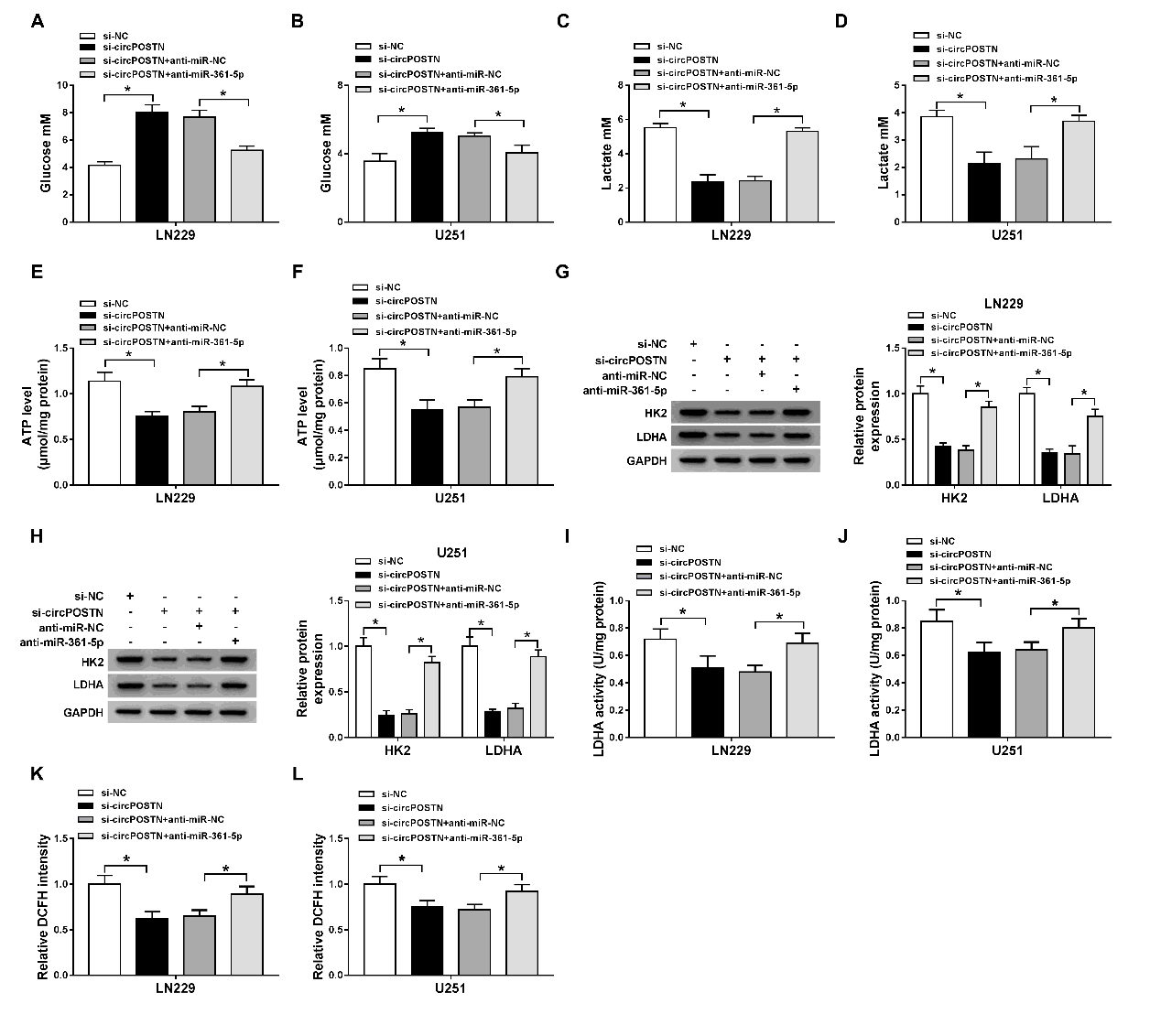

Supplement: Supplementary file 6 — Additional file 6. CircPOSTN silencing inhibited aerobic glycolysis of glioma cells via regulating miR-361-5p. [file 12935_2020_1454_MOESM6_ESM.png]

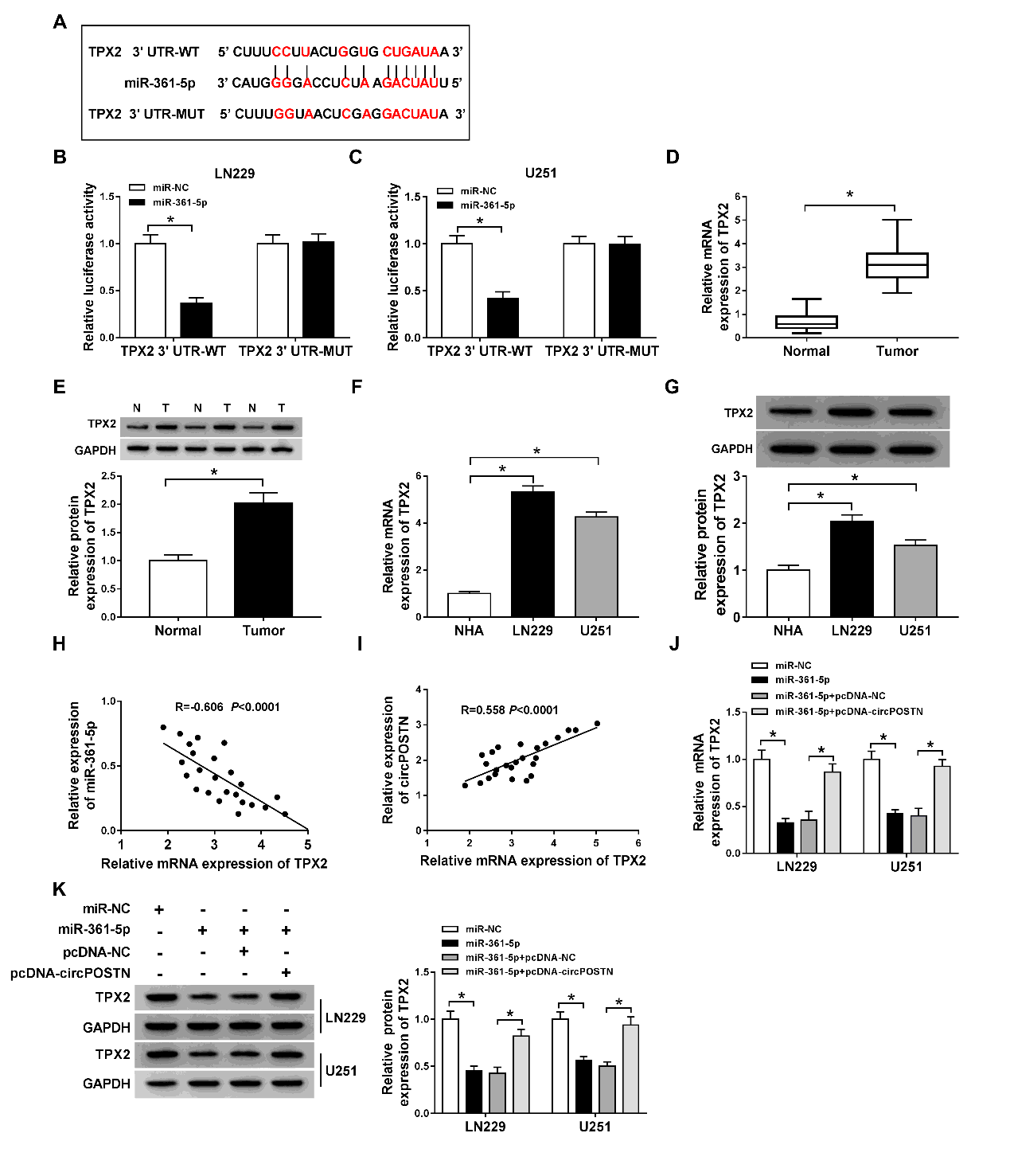

Supplement: Supplementary file 7 — Additional file 7. CircPOSTN regulated TPX2 expression via sponging miR-361-5p in glioma cells. [file 12935_2020_1454_MOESM7_ESM.png]

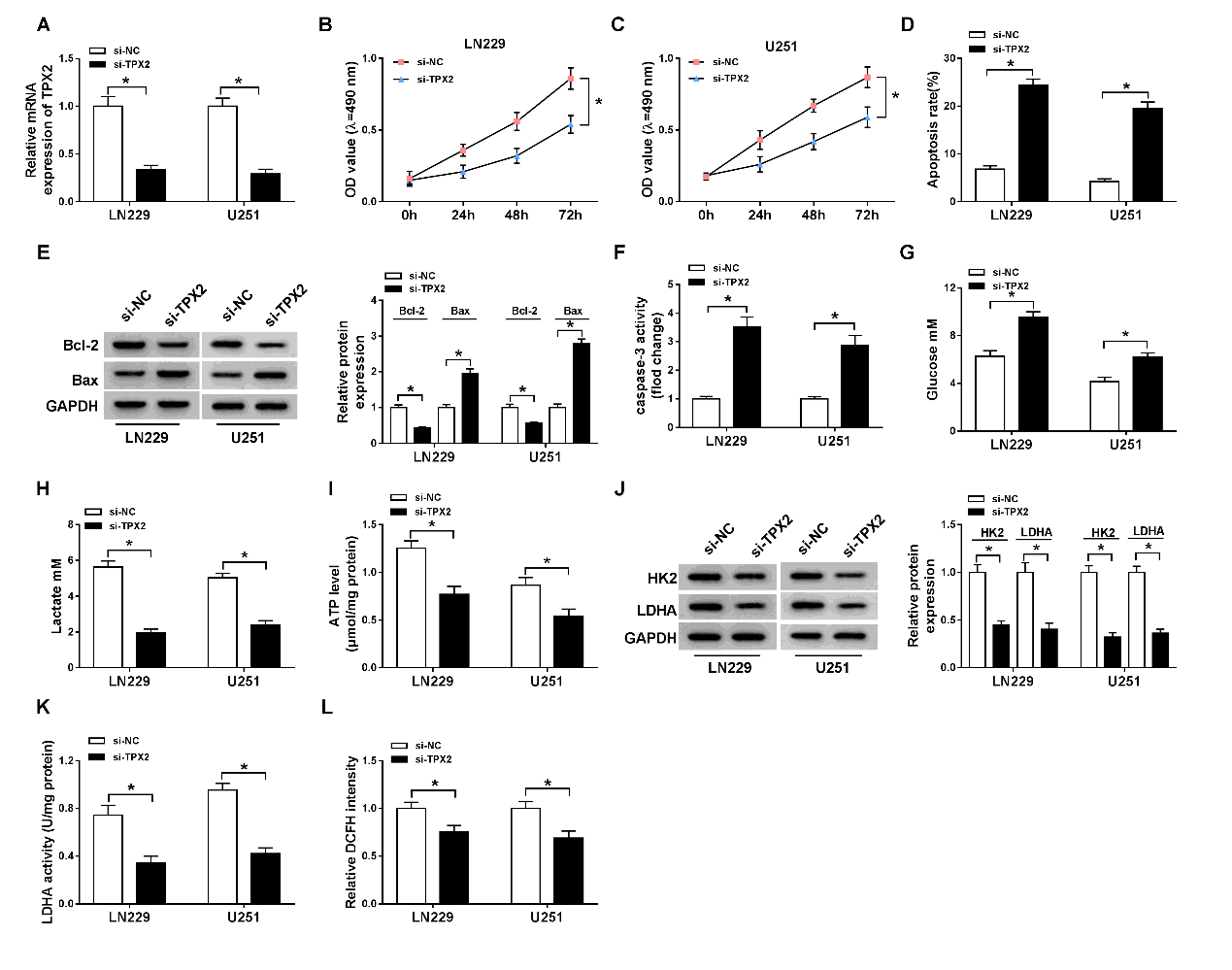

Supplement: Supplementary file 8 — Additional file 8. TPX2 regulated proliferation, apoptosis, and aerobic glycolysis in glioma cells. [file 12935_2020_1454_MOESM8_ESM.png]

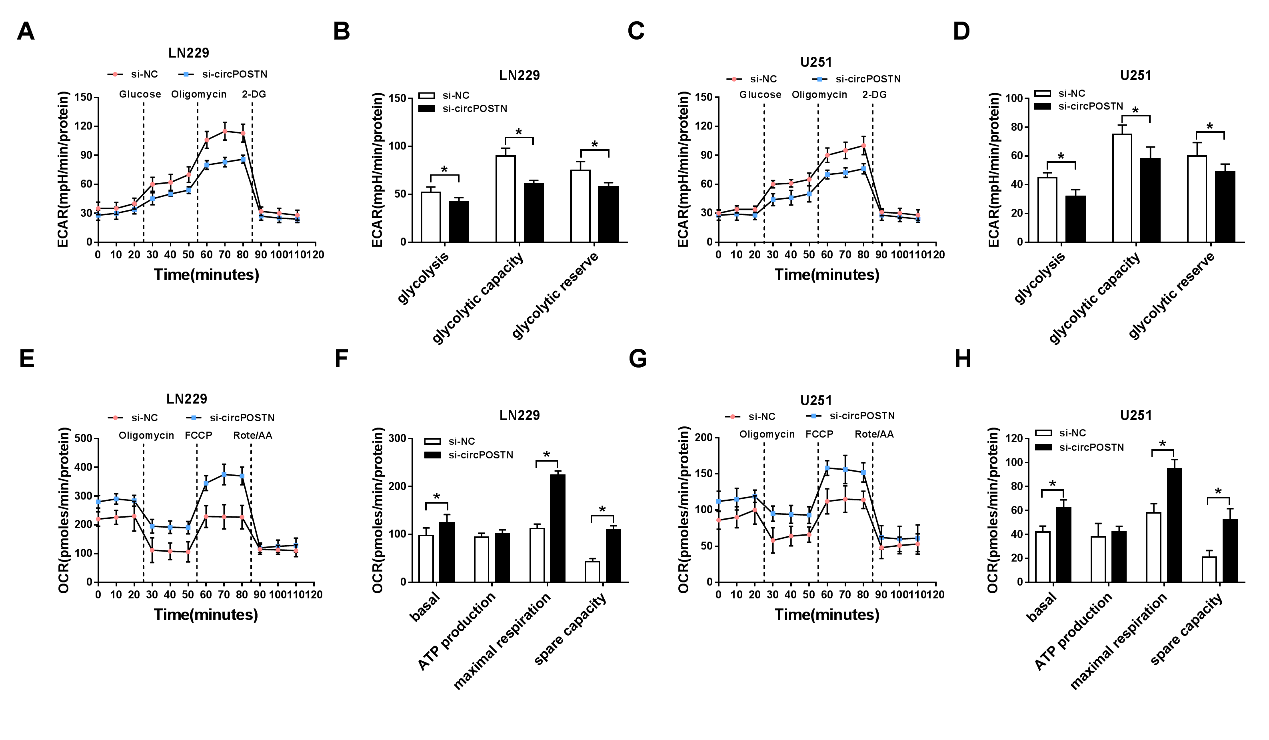

Supplement: Supplementary file 9 — Additional file 9. Extracellular acidification rate and oxygen consumption rate assays in glioma cells. [file 12935_2020_1454_MOESM9_ESM.png]

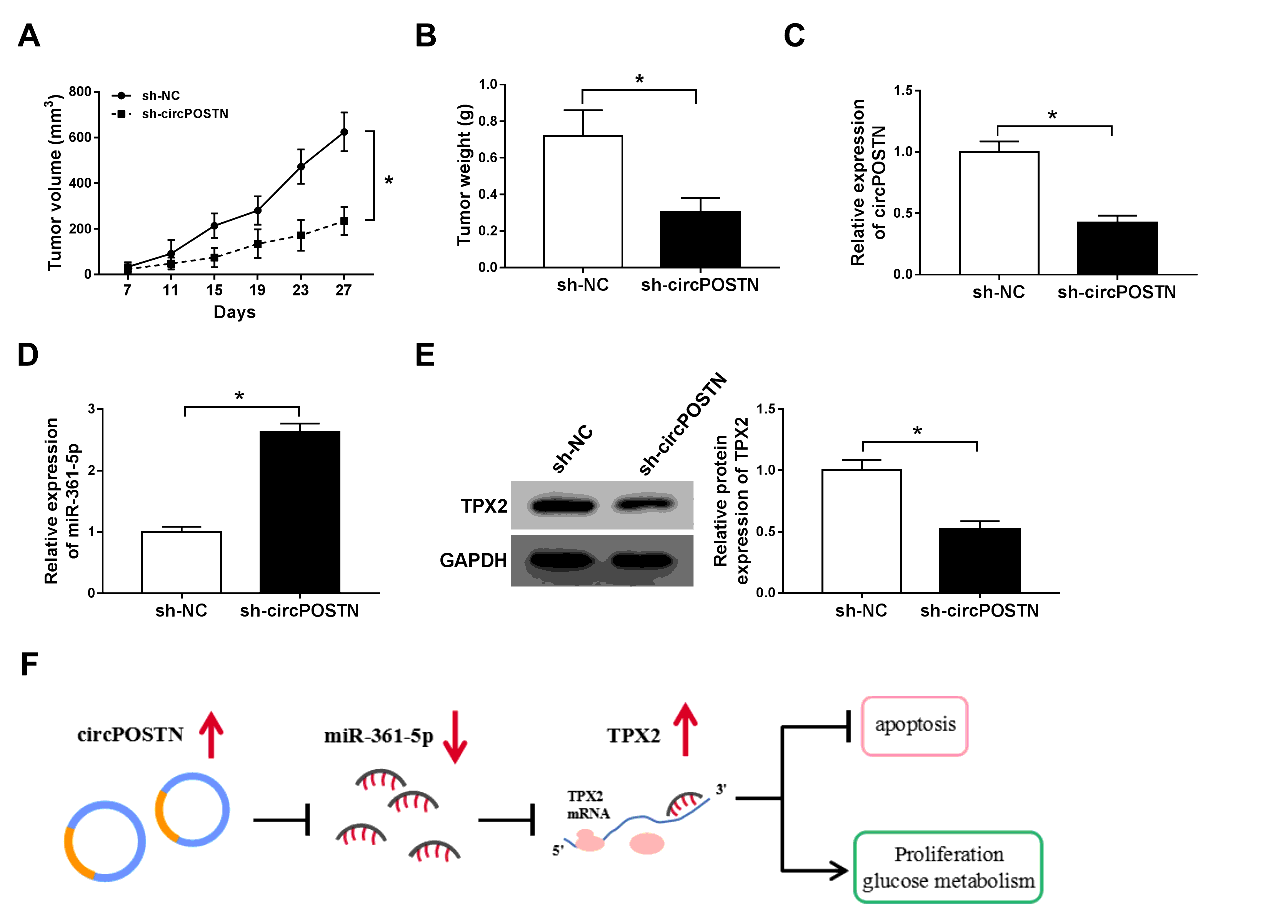

Supplement: Supplementary file 10 — Additional file 10. Silencing of circPOSTN repressed glioma tumor growth in vivo. [file 12935_2020_1454_MOESM10_ESM.png]
